# Risks of uterus transplantation: a scoping review

**DOI:** 10.61622/rbgo/2026rbgo14

**Published:** 2026-03-20

**Authors:** Sibele Maria Schuantes-Paim, Dani Ejzenberg, Beatriz Souza Braga Mergulhão, Gabriela Mininel de Medeiros, Anne Caroline Neves Geraldo, Vinícius Temóteo Ferrari, Bartira de Aguiar Roza, Janine Schirmer

**Affiliations:** 1 Universidade Federal de São Paulo São Paulo SP Brazil Universidade Federal de São Paulo, São Paulo, SP, Brazil.; 2 Universidade de São Paulo Hospital das Clínicas Faculdade de Medicina São Paulo SP Brazil Hospital das Clínicas, Faculdade de Medicina, Universidade de São Paulo, São Paulo, SP, Brazil.

**Keywords:** Transplantation, Vascularized composite allotransplantation, Patient safety, Uterus, Nursing

## Abstract

**Objective:**

To map risks related to uterus transplantation.

**Methods:**

Scoping review based on the Joanna Briggs Institute method. Were included studies published in scientific journals that described complications, adverse events, and risks in women who were uterine transplant recipients, living uterine donors, and newborns, infants and children born from transplanted uteri. A modified version of Failure Modes and Effects Analysis was used for data collection, and the data were quantitatively analyzed through the development of risk categories and risks rates calculation.

**Results:**

In total 44 studies from 13 countries were included. Mapped 115 patients, 62 recipients, 34 donors, and 19 newborns, infants, and children born from transplanted uteri, 324 complications and adverse events across 25 risk categories. An online system (UTx Observatory) was developed. Among recipients, 77.5% received uteri from living donors, and 22.5% from deceased donors with most prevalent immunological, vascular, infectious, and prematurity risks. Recipients of deceased donor uteri had a higher adjusted risk rate. Among living donors, most prevalent risks were neurological, infectious, and urological risks. Laparotomy (76.5%) was associated with a higher adjusted risk rate than robotic-assisted surgery (23.5%) for uterus donation. Mainly risks mapped from newborns, infants, and children were respiratory, development-related, and malformations.

**Conclusion:**

The study and the UTx Observatory provide a foundation for developing targeted risk-mitigation strategies, shaping future studies and interventions, and informing health teams, authorities, and patients involved in uterus transplantation.

Open Science Framework (OSF): https://doi.org/10.17605/OSF.IO/524UT

## Introduction

Uterus transplantation (UTx) is an infertility treatment that enables women diagnosed with absolute uterine factor infertility (AUFI) to experience pregnancy and childbirth. AUFI can arise from congenital conditions, such as Mayer-Rokitansky-Küster-Hauser syndrome, or acquired conditions, including hysterectomy due to malignancies or severe bleeding disorders.^(1,2)^

The UTx process is complex and involves screening both the donor and the recipient. The donor can be either living or deceased, and the screening and follow-up procedures differ in each case. After donor and recipient selection, the process begins with ovarian stimulation and oocyte retrieval, as well as sperm collection from the partner, to generate embryos. These embryos are cryopreserved. The process continues with two surgical procedures: the removal of the uterus from the donor and its transplantation into the recipient. This is followed by close monitoring of both the living donor and the recipient, as well as evaluation of graft viability. Immunosuppressive therapy is required to prevent graft rejection and ensure the long-term functionality of the transplanted uterus, with protocols specifically tailored to future pregnancy. After the transplant, the recipient typically begins menstruating. Following an appropriate healing period, embryo transfers are initiated, one embryo at a time. Pregnancies achieved after UTx are considered risk pregnancies, requiring cesarean delivery, with subsequent permanent removal of the transplanted uterus after childbirth.^(3,4)^

Despite recent advances, research on UTx conducted by healthcare teams and social researchers, points to the limited knowledge about the risks involved in uterine transplantation underscoring the importance of systematically mapping complications and adverse events to enhance patient safety. Patient safety is defined as "the reduction of risk of unnecessary harm associated with healthcare to an acceptable minimum," considering current knowledge and available resources.^(5,6)^

Therefore, understanding the risks associated with UTx is important since authors question whether UTx is appropriate, given the potential for harm not only to recipients but also to living donors and to newborns, infants, and children born from transplanted uteri.^(6)^ The ethical debate surrounding this infertility treatment underscores the need for a holistic and transparent appraisal of the risks involved, especially now that UTx has become a viable option for women with AUFI in some countries.^(7)^

Therefore, studying the risks linked to health procedures is a key to a safer care, understanding its hazards and creating targeted tools can guide public policy and improve clinical decisions. This scoping review aims to map risks related to UTx.

## Methods

The scoping review followed the Joanna Briggs Institute (JBI) method, suitable for mapping evidence, identifying knowledge gaps, and building a conceptual framework, especially in emerging fields with complex and heterogeneous literature.^(8)^

The study began in January 2024 with a preliminary search for existing or ongoing protocols, systematic reviews, and scoping reviews on the same subject and proposed analysis, and as far as could be assessed, no other review or protocol had been published or was under development.

Subsequently, the review protocol was developed in March 2024, registered on 16 August 2024 on the Open Science Framework (OSF) platform, updated and published on 21 May 2025.^(9)^

Research question, objective, title, and inclusion criteria were delineated based on the PCC mnemonic strategy (Population, Concept, Context): Population: women who have undergone UTx, irrespective of infertility etiology, donor type, immunosuppression regimen, or pregnancy status/outcome; living uterine donors; and newborns, infants, and children born from transplanted uteri; concept: complications, adverse events, and risks; and context: any healthcare setting (outpatient or inpatient) in any country.

The study concept comprises three terms: complications, adverse events, and risks.

- Complications: problems occurring during or after a procedure or treatment.^(10)^ Specifically associated with donation (donor hysterectomy), transplantation, cesarean birth, and hysterectomy to recipient, including living donor and newborns, infants, and children from transplanted uteri.
- Adverse events: undesirable and unexpected occurrences related to any stage of the procedural chain.^(5)^ From donation (donor hysterectomy), transplantation, cesarean birth, and hysterectomy to recipient capable of causing harm to recipients, living donors, or newborns, infants, and children from transplanted uteri.
- Risks: potential hazards with a probability of causing harm.^(10)^

Clinical trials, prospective and retrospective observational studies, case reports and case series, commentaries, and videos published in scientific journals were included. Was not included literature reviews (systematic, integrative, narrative, or scoping) or conference abstracts, in vitro studies, and animal model studies.

These literature types were excluded because in vitro or animal model research may yield results that differ significantly from those obtained in humans, potentially compromising representativeness and biasing the conclusions of the analysis. The exclusion of literature reviews and conference abstracts was justified by the risk of data duplication, which could also lead to incorrect interpretations of the findings.

No language restrictions were applied, but only studies from 2014 onward were included, due to updated biovigilance definitions and the first live birth after UTx occurring that year, marking significant advancements in the field.

Search strategies were developed in three stages: preliminary search, comprehensive search, and backward- and forward-citation searching of the included records. For the first stage, the preliminary search, general UTx-related terms were applied in the PubMed and CINAHL databases to refine keywords and identify the most suitable indexing terms. The second stage, the comprehensive search, was performed in four databases and one electronic library (PubMed, CINAHL, Scopus, Web of Science, and SciELO) using specific strategies in May 2024 and updated in May 2025.

The comprehensive search for the scoping review:

- PubMed: ((("uterus transplantation"[All Fields]) OR ("uterus transplant"[All Fields])) OR ("womb transplant"[All Fields])) AND ("outcomes"[All Fields]) – last 10 years.
- CINAHL: uterus transplantation OR uterus transplant AND adverse effects OR side effects OR negative effects OR complication OR risk – last 10 years.
- SCOPUS: uterus AND transplantation AND outcomes – last 10 years.
- SciELO: (uterus transplantation) OR (uterus transplant) – last 10 years.
- Web of Science: uterus transplantation (All Fields) AND outcomes (All Fields) OR clinical outcomes – last 10 years.

Finally, a backward- and forward-citation search was conducted through an analysis of the reference lists of the articles to identify additional materials that might not have been captured by the original search strategies. In addition, a supplementary search was performed on the ClinicalTrials.gov platform to locate publications related to UTx clinical trials that had not been retrieved previously. These two final steps were carried out after the initial screening of studies, in September 2024, and at the end of the update in May 2025 to ensure that no relevant publication had been omitted owing to the limited sensitivity of the original search strategies. Consequently, 49 additional records were identified.

The identified articles were exported to the Rayyan platform, where the research team carried out the initial screening by reading titles and abstracts between June and September 2024, and in May and June 2025. This screening was conducted independently and blinded by each researcher. Subsequently, any conflicts in decisions were assessed separately to reach consensus.

Full-text articles were exported to Zotero and independently reviewed (Sept 2024–June 2025) for inclusion criteria. Disagreements were resolved through detailed reassessment, resulting in full consensus.

After the selection of the articles that composed the review had been completed, data collection was initiated. To assist this process, a Microsoft Excel spreadsheet was structured to record the following information: authorship; country of the study; year of publication; year of data collection; objective; study design; population and sample; patient type (recipient, living donor, or newborn, infant or child); complications or adverse events; associated signs and symptoms; risk factors; investigative process (indicators and tests requested, procedures for determining severity and the correlation); management and follow-up strategies; and reference. Additional information considered relevant by the researchers was also recorded under "notes." Data collection began in September 2024 and was completed in July 2025.

Initially, all complications and adverse events described were grouped into specific categories termed "risks," based on their diagnostic and clinical similarity. These risks were then allocated according to the phases of the UTx process, ensuring a clear analysis of the temporality of the occurrences.

Next, the identification of the patients mentioned in the studies was standardized, clearly distinguishing transplant recipients, donors, and newborns, infants or children born from transplanted uteri. Accordingly, the risk categorization was structured specifically for each group. Because all articles provided sufficient descriptions, it was possible to correlate cases across studies and to link each patient with all related risks.

This analysis was possible because it was conducted using the modified Failure Mode and Effects Analysis (mFMEA). This version begins with human anatomy and physiology and estimates risks from detailed descriptions obtained in case reports.^(11)^ This framework made it possible to conduct a scientifically precise analysis, allowing for the determination of risk categories and the correlation of the data.

After the risk categories had been established and patient information standardized, statistical analyses were performed, including the absolute (n) and relative frequencies (%) of the identified risks. In addition, the risk rate per group was calculated based on exposure, yielding the rate of complications and adverse events (risks) per 100 patients. The formula used was:


$$\text{Risk rate}=\frac{\begin{array}{c} \text{Number of complications and}\\ \text{adverse events} \end{array}}{\text{Number of exposed patients}}\times 100$$

Quantitative analyses were performed using the Python programming language on the Cursor platform, with the support of the pandas and seaborn libraries for graphical visualization of the results. All data analysis was carried out between May and August 2025.

The review data are presented through textual descriptions, tables, graphs, and an online system, the UTx Observatory. This system provides the entire database, relevant descriptions, and an analytical dashboard showing the review's findings. Results were reported in accordance with the Preferred Reporting Items for Systematic Reviews and Meta-Analyses extension for Scoping Reviews (PRISMA-ScR).^(12)^

Approval from a research ethics committee was not required.

## Results

**Figure 1:**
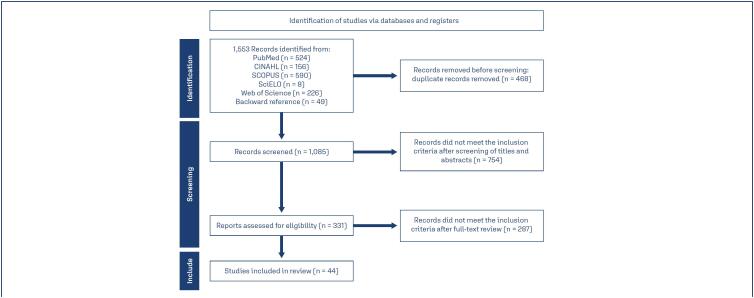
Selection workflow for articles in the scoping review

A total of 1,553 articles were identified, and of these, 44 were included in the study; details of the article-selection process are presented in figure 1.

Were included studies published between 2014 and 2025, with at least one study published each year. The years with the highest number of publications were 2020 (n = 9; 21%), 2023 (n = 6; 14%), and 2021 (n = 5; 11.7%). Articles originated from 13 different countries, with two transplant centers located in the United States – Baylor University Medical Center and Cleveland Clinic. It is known that transplant programs exist in other transplant centers in the United States; however, only in these centers was it possible to identify articles that met the inclusion criteria of the review. The countries with the greatest volume of publications were the United States (n = 15; 34.1%), Sweden (n = 10; 22.7%), and the Czech Republic (n = 5; 11.4%). Detailed information, including country of origin, authorship, study design, and the titles of all included studies, is provided in chart 1.

**Chart 1:** Characterization of the articles included in the review by country of origin, year of publication, authorship, study design, and title (n=44)

Country (Year)	Authorship and study design	Title
Sweden (2014)	Brännström et al.^(13)^ Study design: Prospective observational study.	First clinical uterus transplantation trial: a six-month report.
Sweden (2014)	Brännström et al.^(14)^ Study design: Case report.	Livebirth after uterus transplantation.
Sweden (2015)	Johannesson et al.^(15)^ Study design: Prospective observational study.	Uterus transplantation trial: 1-year outcome.
Sweden (2016)	Brännström et al.^(16)^ Study design: Case report part of an observational study.	One uterus bridging three generations: first live birth after mother-to-daughter uterus transplantation.
Sweden (2017)	Kvarnström et al.^(17)^ Study design: Observational study.	Live donors of the initial observational study of uterus transplantation-psychological and medical follow-up until 1 year after surgery in the 9 cases.
United States (2017)	Testa et al.^(18)^ Study design: Clinical trial.	Living donor uterus transplantation: a single left's observations and lessons learned from early setbacks to technical success.
United States (2017)	Flyckt et al.^(19)^ Study design: Video case report.	Deceased donor uterine transplantation.
China (2017)	Wei et al.^(20)^ Study design: Case report.	Modified human uterus transplantation using ovarian veins for venous drainage: the first report of surgically successful robotic-assisted uterus procurement and follow-up for 12 months.
Czech Republic (2019)	Chmel et al.^(21)^ Study design: Clinical trial.	Revaluation and lessons learned from the first 9 cases of a Czech uterus transplantation trial: Four deceased donor and 5 living donor uterus transplantations.
United States (2018)	Testa et al.^(22)^ Study design: Case report.	First live birth after uterus transplantation in the United States.
Brazil (2019)	Ejzenberg et al.^(23)^ Study design: Case report.	Livebirth after uterus transplantation from a deceased donor in a recipient with uterine infertility.
India (2018)	Puntambekar et al.^(24)^ Study design: Case report.	Laparoscopic-assisted uterus retrieval from live organ donors for uterine transplant: our experience of two patients.
Czech Republic (2019)	Kristek et al.^(25)^ Study design: Case report.	Acute appendicitis in a patient after a uterus transplant: a case report.
United States (2019)	Johannesson et al.^(26)^ Study design: Commentary.	Rethinking the time interval to embryo transfer after uterus transplantation – DUETS (Dallas UtErus Transplant Study).
United States (2020)	Ramani et al.^(27)^ Study design: Cases report.	DUETS (Dallas UtErus Transplant Study): complete report of 6-month and initial 2-year outcomes following open donor hysterectomy.
United States (2020)	Testa et al.^(28)^ Study design: Cases report.	The evolution of transplantation from saving lives to fertility treatment: DUETS (Dallas UtErus Transplant Study).
Sweden (2020)	Brännström et al.^(29)^ Study design: Cases report.	Outcome of recipient surgery and 6-month follow-up of the Swedish live donor robotic uterus transplantation trial.
Sweden (2020)	Brännström et al.^(30)^ Study design: Prospective observational study.	Evolution of surgical steps in robotics-assisted donor surgery for uterus transplantation: results of the eight cases in the Swedish trial.
Sweden (2020)	Brännström et al.^(31)^ Study design: Case report.	Live birth after robotic-assisted live donor uterus transplantation.
United States (2020)	Flyckt et al.^(32)^ Study design: Case report.	First birth from a deceased donor uterus in the United States: from severe graft rejection to successful cesarean delivery.
Germany (2020)	Brucker et al.^(33)^ Study design: Prospective observational study.	Living-donor uterus transplantation: pre-, intra-, and postoperative parameters relevant to surgical success, pregnancy, and obstetrics with live births.
Spain (2021)	Carmona et al.^(34)^ Study design: Case report.	Uterine transplantation. First viable case in Southern Europe.
China (2020)	Huang et al.^(35)^ Study design: Case report.	Report of the first live birth after uterus transplantation in People's Republic of China.
Lebanon (2020)	Akouri et al.^(36)^ Study design: Case report.	First live birth after uterus transplantation in the Middle East.
Czech Republic (2021)	Fronek et al.^(37)^ Study design: Clinical trial.	Human uterus transplantation from living and deceased donors: the interim results of the first 10 cases of the Czech trial.
Czech Republic (2021)	Fronek et al.^(38)^ Study design: Case report.	Live birth following uterine transplantation from a nulliparous deceased donor.
United States (2021)	Johannesson et al.^(39)^ Study design: Comparative study.	Dallas UtErus Transplant Study: early outcomes and complications of robot-assisted hysterectomy for living uterus donors.
United States (2021)	Rosenzweig et al.^(40)^ Study design: Case report.	Pregnancy after CMV infection following uterus transplantation: a case report from the Dallas Uterus Transplant Study.
United States (2021)	Johannesson et al.^(41)^ Study design: Prospective study reports.	Twelve live births after uterus transplantation in the Dallas UtErus Transplant Study.
Sweden (2022)	Brännström et al.^(42)^ Study design: Prospective study.	Reproductive, obstetric, and long-term health outcome after uterus transplantation: results of the first clinical trial.
Sweden (2022)	Karlsson et al.^(43)^ Study design: Cases report.	Hysterectomy after uterus transplantation and detailed analyses of graft failures.
United States (2022)	Schulz et al.^(44)^ Study design: Cohort study.	Children after uterus transplantation: 2-year outcomes from the Dallas UtErus Transplant Study (DUETS).
France (2022)	Ayoubi et al.^(45)^ Study design: Case report.	Case report: post-partum SARS-CoV-2 infection after the first French uterus transplantation.
Czech Republic (2023)	Janota et al.^(46)^ Study design: Observational study.	Three-year follow-up results of two children born from a transplanted uterus.
United States (2023)	Wilson et al.^(47)^ Study design: Cases report.	Immunosuppression in uterus transplantation: experience from the Dallas Uterus Transplant Study.
United States (2023)	York et al.^(48)^ Study design: Prospective study.	Neonatal outcomes after uterus transplantation: Dallas Uterus Transplant Study.
Australia (2023)	Deans et al.^(49)^ Study design: Case report.	The first Australian uterus transplantation procedure: a result of a long-term Australian-Swedish research collaboration.
Australia (2023)	Georgevsky et al.^(50)^ Study design: Case report.	First uterine transplant case at the Royal Prince Alfred Hospital.
Italy (2023)	Scollo et al.^(51)^ Study design: Case report.	Live birth from cryopreserved oocyte after uterus transplantation: a case report.
United States (2024)	D’Amico et al.^(52)^ Study design: Case report.	Uterus transplantation: a rescue technique to save the viability and functionality of the graft after intra-operative outflow thrombosis.
Italy (2024)	Veroux et al.^(53)^ Study design: Case report.	Uterus transplantation from deceased donors: first Italian experience.
United States (2024)	Jacques et al.^(54)^ Study design: Prospective study.	Robotic living donor hysterectomy for uterus transplantation: an update on donor and recipient outcomes.
Singapore (2025)	Tan et al.^(55)^ Study design: Case report.	Living donor uterus transplant research project in Singapore: Progress of the first case.
Australia (2025)	Deans et al.^(56)^ Study design: Case report.	The first live term birth following uterus transplantation in Australia.

From the analysis of the 44 articles, 324 occurrences of complications and adverse events were identified. These findings were grouped into 25 risk categories, established according to diagnostic and clinical similarity. An online system has been developed that systematizes all the data from the review. The *UTx Observatory* offers open access to the complete database as well as to every analysis presented in this article through an interactive dashboard. As new studies emerge, additional risks, complications, or adverse events can be incorporated into the platform, thereby ensuring that the risk assessment for UTx remains accurate and up to date. The platform can be accessed at: utxobservatory.org. The risk classification based on the complications and adverse events reported in the literature are presented in chart 2.

**Chart 2:** Risk classification for uterus transplantation based on the complications and adverse events described in the literature and grouped according to clinical and diagnostic similarity

Risk categories	Complications and adverse events
Amniotic fluid disorder risk	Recipient: polyhydramnios; oligohydramnios.^(33,41)^
Fetal loss risk	Recipient: septic abortion; intrauterine fetal death.^(28,37)^
Gastrointestinal risk	Recipient: nausea; vomiting; diarrhea; perforated acute appendicitis; adhesions; rectal injury.^(47,55)^
Gestational vascular disorder risk	Recipient: pre-eclampsia; gestational hypertension; disorders of uteroplacental vascularization.^(14,35,37,41,42,45-47)^
Hematological risk	Recipient: anemia; leukopenia; reduced hemoglobin levels.^(14,16,28,36-38,43,49)^ Living donor: anemia; reduced hemoglobin levels.^(27,28,50)^ Newborn: anemia.^(48)^
Hepatic risk	Recipient: intrahepatic cholestasis; hepatotoxicity; impaired hepatic function.^(16,33,42,47)^
Hormonal risk	Recipient: ovarian hyperstimulation syndrome.^(21,45)^ Living donor: climacteric symptoms.^(21,37)^ Child: idiopathic premature thelarche.^(46)^
Immunological risk	Recipient: mild, moderate, severe, prolonged, and borderline rejection; donor-specific antibody (DSA) reaction.^(13-16,20,21,25,26,32-34,37,47)^
Infectious risk	Recipient: graft failure caused by infection; Enterococcus faecium infection detected in vaginal culture; surgical-site infection; Enterococcus faecalis, Enterobacter, and Klebsiella pneumoniae infections; cytomegalovirus (CMV) infection; urinary tract infection; Clostridioides difficile infection (colitis); Epstein–Barr virus (EBV) infection; herpes simplex virus-2 (HSV-2) infection; sinusitis; mastitis; cervicitis; pelvic abscess; incisional erythema; Candida vascular infection; upper respiratory tract viral infection; atypical vaginal discharge; pyelonephritis; SARS-CoV-2 infection; Aspergillus and Pneumocystis pneumonia.^(13,19 21,23,25,27,28,33,36,37,40,43,45,47,51,53,55)^ Living donor: surgical-site infection; pyelonephritis; urinary tract infection; Clostridioides difficile urinary tract infection; Escherichia coli infection and sepsis; elevated C-reactive protein levels; upper respiratory tract infection.^(17,18,27,28,30,36,45,55)^ Newborn, infant, and child: bandemia, recurrent ear infections, monthly upper respiratory tract infection symptoms; SARS-CoV-2 infection.^(44,45,48)^
Mental health–related risk	Living donor: depression.^(18,27,28)^
Metabolic disorder risk in pregnancy	Recipient: gestational diabetes mellitus.^(28,37,41,46,47)^
Metabolic risk in the newborn	Newborn: hypoglycemia; neonatal jaundice.^(33,48)^
Neurological risk	Recipient: neurotoxicity; inguinal pain.^(33)^ Living donor: meralgia paresthetica; gluteal claudication with ambulation; dyspareunia; transient peroneal nerve palsy; temporary paresis of the lateral sural cutaneous nerve; fecal impaction.^(17,18,27,28,31,37)^
Placental disorder risk	Recipient: placenta previa; retained placenta; central placenta previa with accreta.^(28,32,37,41)^
Prematurity risk	Recipient: premature contractions; preterm labor; preterm birth; cervical insufficiency; reduced cervical length; premature rupture of membranes; vaginal bleeding during pregnancy.^(26,28,31,33,35,36,40,41,45,54,56)^
Renal risk	Recipient: hydronephrosis; chronic kidney disease; impaired renal function; nephrotoxicity; acute renal failure.^(14,16,26,28,32,37,41,45,47)^ Living donor: hydronephrosis.^(33)^
Respiratory risk	Recipient: pleural effusion; pneumothorax.^(13,28)^ Living donor: pulmonary atelectasis; prolonged intubation.^(28,36)^ Newborn, infant, and child: mild respiratory distress; mild respiratory distress syndrome; bronchiolitis; tachypnea; mild acute respiratory distress syndrome and retained pulmonary fluid; apnea of prematurity; central cyanosis due to respiratory distress syndrome; respiratory maladaptation.^(31,33,36,46,48,56)^
Risk of hypothermia	Newborn: hypothermia.^(33)^
Risk of malformations	Newborn: auricular deformity; clitoromegaly; displaced urethra; cryptorchidism; bilateral inguinal hernia; unilateral anechoic adrenal cyst.^(44,45,48)^
Risk related to immunosuppressant exposure	Recipient: moderate squamous epithelial dysplasia.^(15)^ Newborn: elevated serum level of tacrolimus.^(35)^
Risk related to newborn, infant and child development	Newborn, infant, and child: mild expressive communication deficit; delayed communication development; growth below expectations; low birth weight.^(44,46,48)^
Risk related to prolonged surgical positioning	Living donor: pressure alopecia.^(28,30,39)^
Surgical risk of vaginal anastomosis	Recipient: vaginal stenosis.^(21,29,31,34,36-38,53)^ Living donor: vaginal-cuff dehiscence.^(18,27,28,54)^
Urological risk	Recipient: vesicovaginal fistula; bladder injury.^(21,28)^ Living donor: nocturia; uterovaginal fistula; partial ureteral laceration; hypotonic urinary bladder; urinary retention; thermal injury to the urethra; distal ureteral injury.^(13,17,21,28,37,39,45,49)^
Vascular risk	Recipient: retroperitoneal hematoma; infected vaginal-cuff hematoma; graft failure caused by thrombosis; vaginal hematoma; thrombosis; hematoma; hemorrhage; subchorionic hematoma; vaginal bleeding; right lower-limb ischemia; perfusion problems; hemorrhagic shock; absence of arterial flow; hematuria; severe hemorrhage; hemoperitoneum; vaginal bleeding after biopsy.^(13,16,18,21,23,24,28,29,31-33,35,37,41,52,53)^ Living donor: hemorrhage; graft not used because of thin vasculature; lower-limb edema; hydronephrosis due to a ureteral blood clot; hematuria.^(17,27,28,37,39,55)^

Moreover, the stages of the UTx process were classified using the case descriptions, encompassing donor hysterectomy, transplantation in the recipient, pregnancy, cesarean birth, recipient hysterectomy, and follow-up of the living donor, recipient, and newborn, infant, and child.

- Uterus Donation - Hysterectomy in the donor: Perioperative of hysterectomy in the donor; Immediate postoperative period (up to 24 hours); Early postoperative period (24 hours to 7 days); Late postoperative period (7 days to 29 days); Postoperative period 30-90 days (1 to 3 months); Postoperative period 91-180 days (3 to 6 months); Postoperative period 181-365 days (6 to 12 months).
- In Vitro Fertilization (IVF): Oocyte retrieval protocol and embryo generation before UTx and Embryo Transfer after UTx.
- Uterus Transplantation in the recipient: Perioperative of transplantation in the recipient; Immediate postoperative period (up to 24 hours); Early postoperative period (24 hours to 7 days); Late postoperative period (7 days to 29 days); Postoperative period 30-90 days (1 to 3 months); Postoperative period 91-180 days (3 to 6 months); Postoperative period 181-365 days (6 to 12 months); Postoperative period 366-730 days (12 to 24 months); Postoperative period 731-1096 days (24 to 36 months).
- Pregnancy: First trimester of pregnancy (1 to 13 weeks); Second trimester of pregnancy (14 to 26 weeks); Third trimester of pregnancy (27 to 40 weeks).
- Cesarean birth: Perioperative of cesarean in the recipient (birth); Immediate postoperative period (up to 24 hours); Early postoperative period (24 hours to 7 days); Late postoperative period (7 days to 29 days); Postoperative period 30-90 days (1 to 3 months).
- Hysterectomy after birth: Perioperative of hysterectomy in the recipient (hysterectomy after birth); Immediate postoperative period (up to 24 hours); Early postoperative period (24 hours to 7 days); Late postoperative period (7 days to 29 days); Postoperative period 30-90 days (1 to 3 months).
- Brith and monitoring of newborn, infant and child after UTx: Peripartum (cesarean); Immediate postpartum (up to 24 hours); Newborn (24 hours to 30 days); Infant 30-90 days of life (1 to 3 months); Infant 91-180 days of life (3 to 6 months); Infant 181-365 days of life (6 to 12 months); Child 366-730 days of life (12 to 24 months); Child 731-1096 days of life (24 to 36 months).

It should be emphasized that, in this review, the uterine donation and transplantation processes begins with the donor's hysterectomy and the graft implantation in the recipient. Issues of eligibility related to the selection processes for living donors and recipients, as well as procedures concerning deceased donors, were not included.

**Figure 2:**
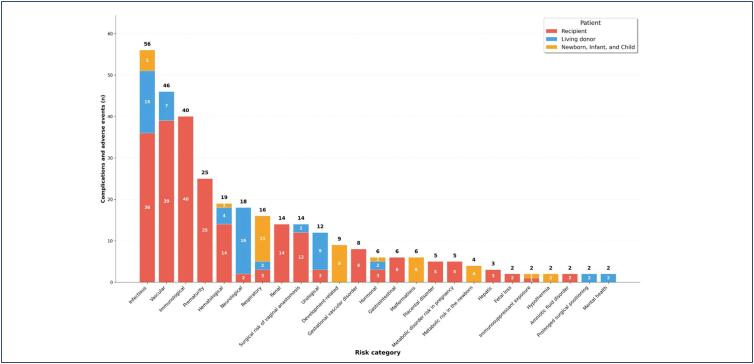
Complications and adverse events by risk category and patient type

Regarding the study population, 115 patients were identified: 62 UTx recipients, with 223 occurrences of complications and adverse events grouped into 19 risk categories; 34 living donors, with 61 occurrences grouped into 10 risk categories; and 19 newborns, infants, and children with 40 occurrences classified into nine risk categories. 16 descriptions of complications or adverse events were excluded because their accurate relationship to a patient could not be determined, thus avoiding duplication.^(16,22,42,43)^Figure 2 displays the absolute frequency of complications and adverse events by risk category and patient type.

Of the 62 recipients identified in the articles, 48 (77.5 %) received a transplant from a living donor, accounting for 167 occurrences of complications and adverse events, among which risks immunological (n = 30; 18%),^(13-16,20,21,25,26,32-34,37,47)^ vascular (n = 27; 16.2%),^(13,16,18,21,23,24,28,29,31-33,35,37,41,52,53)^ prematurity (n = 24; 14,4%),^(26,28,31,33,35,36,40,41,45,54,56)^ infectious (n = 23; 13.8%),^(13,19,21,23,25,27,28,33,36,37,40,43,45,47,51,53,55)^ and renal (n = 11; 6.6%),^(14,16,26,28,32,37,41,45,47)^ were the most prevalent.

**Figure 3:**
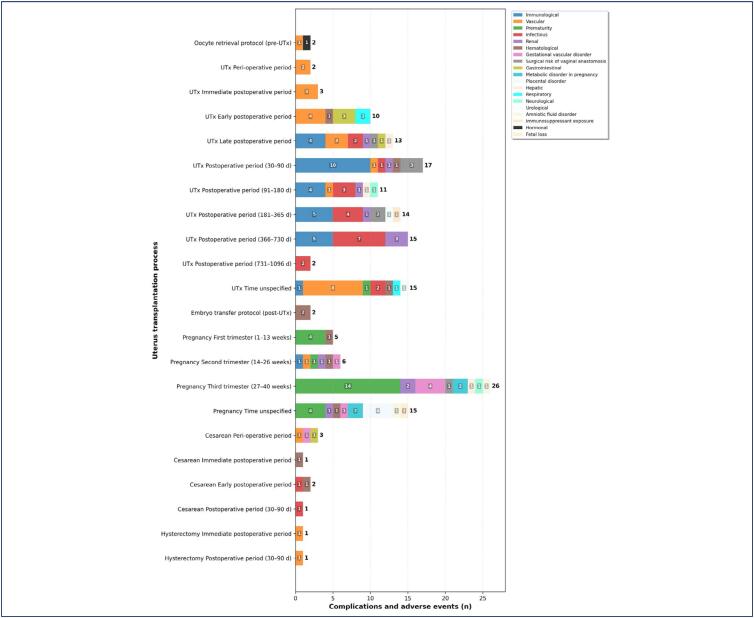
Risks identified in living donor recipients across the different stages of the uterus transplantation process

The timeline (Figure 3) depicts the frequency of these events across the different stages of the transplantation process, illustrating that living donor recipients present more complications at the third gestational trimester (n = 26; 15.6%), 30–90 days UTx postoperative period (n = 17; 10.2%), and 366–730 days (12–24 months) UTx postoperative period (n = 15; 9.0%).

During pregnancy, 52 complications and adverse events were reported, being the most prevalent risk of prematurity (n = 23; 44.3%),^(26,28,31,33,35,36,40,41,45,54,56)^ gestational vascular disorder risk (n = 6; 11.6%),^(14,35,37,41,42,45-47)^, and risks of placental disorder,^(28,32,37,41)^ metabolic disorder in pregnancy^(28,37,41,46,47)^ and renal function^(14,16,26,28,32,37,41,45,47)^ (n = 4; 7.7% each).

This review included prematurity as a complication, only cesarean occurring earlier than scheduled due to factors such as premature membrane rupture or pre-eclampsia. Although prematurity poses neonatal risks, in this study it was classified as a complication related to the gestational phase of UTx, focusing on the recipient's clinical condition. Regarding the immunological risk^(13-16,20,21,25,26,32-34,37,47)^ – rejection – was the most prevalent for living donor recipients, however, during pregnancy only one case was reported (second trimester of pregnancy)^(14)^.

Among deceased donor recipients (n = 14; 22.5%), 56 occurrences of complications and adverse events were recorded, with infectious (n = 13; 23.2%),^(13,19,21,23,25,27,28,33,36,37,40,43,45,47,51,53,55)^ vascular (n = 12; 21.4%),^(13,16,18,21,23,24,28,29,31-33,35,37,41,52,53)^ immunological (n = 10; 17.9%),^(13-16,20,21,25,26,32-34,37,47)^ surgical risk of vaginal anastomosis (n = 5; 8.9%),^(21,29,31,34,36-38,53)^ and hematological risk (n = 4; 7,1%)^(14,16,28,36-38,43,49)^ being the most prevalent.

Regarding the UTx process, these risks were more prevalent at 30–90 days UTx postoperative period (n = 12; 21.4%), early (24 h to 7 days) postoperative period (n = 6; 10.7%), and up to one-year UTx postoperative period (n = 5; 8.9%).

When the risks identified in UTx recipients were analyzed separately according to donor typology – living or deceased – distinct risk profiles emerged. The risk rate per 100 recipients, stratified by donor type, indicates that recipients who received uteri from deceased donors exhibited, on average, a higher risk rate (4.00) than those transplanted with uteri from living donors (3.48).

The most prevalent risk categories identified in living donors were neurological (n = 16; 26.2%),^(17,18,27,28,31,37)^ infectious (n = 15; 24.6%),^(17,18,27,28,30,36,45,55)^ urological (n = 9; 14.8%);^(13,17,21,28,37,39,45,49)^ vascular (n= 7; 11.5%),^(17,27,28,37,39,55)^ and hematological (n = 4; 6.6%).^(27,28,50)^ However, the peak of complications and adverse events occurrence (n = 21; 34,4%) were not possible to determine in the donation process, as articles did not provide sufficient information to assign each complication or adverse event to a specific stage of the donation process. Nonetheless, risks were more prevalent up to seven days (n = 11; 18.0%) after hysterectomy, first month after the donation (n = 8; 13.1%), and the first 24 hours after hysterectomy (n = 7; 11.5%).

Regarding hysterectomy procedures in uterine donors, two distinct approaches were reported: laparotomy (n = 26; 76.5%) and robotic-assisted hysterectomy (n = 8; 23.5%). For donors who were submitted to laparotomy, the most prevalent risks were neurological (n = 15; 57.7%),^(17,18,27,28,31,37)^ infectious (n = 13; 50.0%),^(17,18,27,28,30,36,45,55)^ urological^(13,17,21,28,37,39,45,49)^ and vascular^(17,27,28,37,39,55)^ (n = 6; 23.1% each). For those who were submitted to robotic-assisted hysterectomy, the risks infectious,^(17,18,27,28,30,36,45,55)^ urological^(13,17,21,28,37,39,45,49)^ and related to prolonged surgical positioning^(28,30,39)^ (n = 2; 25.0% each) were the most prevalent. The risk rate for surgical approach was 1.12 for robotic-assisted hysterectomy, and 2.00 for laparotomy.

Forty records of complications and adverse events were identified in newborns, infants, and children born from transplanted uteri. The most prevalent risks were respiratory (n = 11; 27.5%),^(28,36)^ development related (n = 9; 22.5%),^(44,46,48)^ malformations (n = 6; 15.0%),^(44,45,48)^ infectious (n = 5; 12.5%),^(44,45,48)^ and metabolic (n = 4; 10.0%).^(33,48)^A peak in complications and adverse events was observed around the cesarean birth – that is, in the immediate postpartum period (n = 14; 35.0%), peripartum period (n = 7; 17.5%), and the first 30 days of life (n = 5; 12.5%).

## Discussion

The analysis of 11 years of development in UTx reveals a remarkable learning curve and maturation in the field. Our findings demonstrate that time was a key determinant, not only for the technical enhancement of the teams and the consolidation of the exchange of experience, but also for a deeper understanding of the biological responses to the intervention, which have become progressively more manageable.

Regarding UTx related risks, initially, deceased donor recipients exhibited higher risk rates than living donor recipients. Classical studies of kidney transplantation – an analogous setting that includes both living and deceased donation – report similar findings.^(57,58)^ Multiple factors may account for this outcome: graft conditions before implantation, donor age, donor maintenance, and organ preservation measures, all of which contribute to poorer post-transplant outcomes.^(59)^

When discussion living donation versus deceased donation, UTx raises specific ethical questions. For living donation, surgical risks and psychosocial impacts on the donor must be weighed. For deceased donation, issues include uterine retrieval consent, potential harm to other organs, and the risk of transplant tourism. A recent study highlights local disparities – cultural and political – that may jeopardize equitable access to the procedure.^(60)^

Thus, no consensus exists regarding the optimal indications for living or deceased donation.^(61)^ Continued clinical research and the development of culturally sensitive ethical guidelines attuned to each healthcare system and to female representation within each country are imperative.^(62)^

Within uterine donation, the review identified risks specific to living donor: neurological, infectious and urological risks. Donor hysterectomy is a fundamental part, as only retrieval of a viable organ with adequately preserved vascular pedicles allows implantation.^(18,20,24)^

Neurological risks as the most prevalent risk category, are associated to cases of meralgia paresthetica, gluteal claudication with ambulation, dyspareunia, transient peroneal nerve palsy, temporary paresis of the lateral sural cutaneous nerve and fecal impaction. After a hysterectomy patients can experience a range of neurological and functional complications that affect comfort, mobility, sexual activity, and bowel function.^(63)^ Overall, the risks associated with donor hysterectomy align with those of elective hysterectomy described in the literature and do not appear to exceed.^(64)^

Early procedures entailed lengthy operating times and greater exposure to risks, reflecting the teams’ learning curve. Robotic-assisted approaches aim to improve safety by reducing both operative time and organ manipulation.^(24,30,31)^ Furthermore, the increased volume of donor hysterectomies has enabled standardization of prophylactic protocols, antimicrobial and anticoagulant, thereby decreasing infectious and vascular risks.^(65)^

When comparing robotic-assisted surgeries over laparoscopy, no consistent superiority for robotic-assisted across broad sets of procedures were identified.^(66)^ Large observational meta-analyses and pooled studies often detect modest benefits for robotic-assisted approach (e.g., fewer conversions, fewer transfusions, small reduction in some complications) but also consistently find higher hospitalization or procedure costs for robotic-assisted surgery.^(67)^ The choose of surgical approach for hysterectomy for uterus donation need to be carefully evaluated mainly based on benefits of using these approaches for the donor, but also considering costs, hospital structure and surgeons experience.^(68)^ More procedures are needed to confirm the risk profiles described in this review.

For living donor UTx recipients, the three most frequent risks were immunological, vascular and prematurity, but for deceased donor recipients were infectious, vascular and immunological risks.

Immunological rejection remains a major challenge, as immunosuppression must be finely balanced to maintain graft viability without jeopardizing pregnancy. Centers perform routine cervical biopsies and immunological monitoring to detect early rejection.^(69)^ As with other organ transplants, appropriate combinations of immunosuppressive drugs and stringent clinical surveillance are essential for favorable outcomes, ensuring both graft function and the birth of healthy newborns.

The use of immunosuppressants also poses ethical questions and acceptability dilemmas, given potential adverse effects such as increased neoplasia risk, hepatic and renal dysfunction in the recipient, and impacts on pregnancy and the fetus.^(70)^ Studies in other transplanted populations document higher neoplasia incidence associated particularly with chronic mTOR inhibitor use. Systematic laboratory monitoring, dosage adjustments, and substitution of high-risk agents are advised.^(71,72)^ Although the relatively limited duration of immunosuppression in UTx may attenuate these events, prolonged surveillance remains indispensable.

Vascular complications constitute another predominant risk in both living and deceased donor recipients. Such events are common in solid organ transplantation owing to complex vascular suturing. Proposed surgical and perioperative interventions aim to reduce vascular manipulation and enhance hemostasis. While no definitive prophylactic protocol exists, anticoagulation regimens tailored to each patient's coagulation profile have shown efficacy in preventing thrombotic events.^(73)^

The third most prevalent risk for deceased donor recipients was infection. Infection is a well-recognized adverse event in organ transplantation, demanding high vigilance because it can cause severe harm, including graft loss.^(74)^ In deceased donor recipients, infection was the most prevalent risk category. This aligns with kidney transplant studies showing higher infection rates in deceased donor recipients compared with those receiving living donor grafts.^(75)^

The last most prevalent risk category for living donor recipients was prematurity. Comparative studies of pregnancies in women using immunosuppression versus the general population show higher maternal and perinatal complications, including gestational diabetes mellitus, hypertension, pre-eclampsia, and increased cesarean birth rates.^(76)^ Prematurity and low birthweight are likewise more frequent.^(77)^ In contrast, a small series of transplant recipients who underwent assisted reproduction reported favorable outcomes, with term births and no graft impairment.^(78)^

In UTx specifically, findings remain preliminary but confirm the procedure's complexity and heightened gestational risk, nonetheless, cesarean birth in UTx is usually planned between gestational weeks 31 and 37.^(2)^

Few complications were reported for newborns, infants, and children, the most common being respiratory, development-related, and malformations risks. Robust studies on newborns, infants, and children born after UTx remain scarce. In the few cases where developmental risks were noted, all children showed positive progression, with only temporary delays in cognitive milestones.^(44,46,48)^

Identifying specific risk factors for UTx newborns is complex because must consider not only maternal immunosuppression and uterine surgery but also donor organ history, assisted reproduction techniques, and genetic and epigenetic variables of parental gametes.^(79,80)^

Therefore, current evidence does not indicate that UTx increases neonatal risks beyond those observed in cesarean births – namely respiratory complications and infection.^(81)^ Nevertheless, given the recency of initial births, ongoing data collection and monitoring are indispensable to fully elucidate these outcomes.

Analysis of the timing of complications and adverse events reveals the moments in the donation–transplantation process when patients are most likely to be affected by treatment risks. Although reporting bias is possible – since authors may emphasize stages, they consider critical – this study identifies the stages and time points at which women and newborns are most exposed to risks. This analysis supports enhanced monitoring and the development of safer protocols targeted to the most critical moments. Notably, the study also proposes a taxonomy of risks in relation to UTx stages, considering existing recommendations.^(82)^

Despite the rigorous methodology employed, this study has limitations that must be considered when interpreting the results. Data extraction relied on the identification and recognition of cases described in original publications. In this process, information may have been omitted or duplicated, warranting caution in the use and generalization of the findings. For this reason, this is a scoping review rather than a systematic review, underscoring the inherent fragility of extrapolating its results. Moreover, the scarcity of publications and reported procedures, coupled with the fact that relatively few UTx have been performed worldwide to date, limits the statistical power of inference and highlights the need for caution when generalizing the results.

## Conclusion

In summary, the review comprehensively mapped documented risks of uterus transplantation, providing a solid reference for future clinical trials. In addition, this study originated the UTx Observatory to systematize published data on UTx allowing users to navigate and interact with the analyzed data, as well as to incorporate new information from published research. This article and the system can guide the development of targeted risk-mitigation strategies, tailor future studies and interventions, and informing health teams, authorities, and patients involved in uterus transplantation.

## Data Availability

The research data are described in the article presented.
